# Antiviral activity of natural and modified hydrocolloids: main sources, susceptible viruses, structure-activity relationship and mechanisms of action

**DOI:** 10.3389/fnut.2025.1696022

**Published:** 2025-11-12

**Authors:** Cláudia S. G. P. Pereira, M. Carpena, João C. M. Barreira, M. A. Prieto, M. Beatriz P. P. Oliveira

**Affiliations:** 1 LAQV/REQUIMTE, Department of Chemical Sciences, Faculty of Pharmacy, University of Porto, Porto, Portugal; 2 Department of Analytical Chemistry and Food Science, Instituto de Agroecoloxía e Alimentación (IAA) - CITEXVI, Universidade de Vigo, Nutrition and Bromatology Group, Vigo, Spain; 3 Mountain Research Centre (CIMO), ESA, Polytechnic Institute of Bragança, Campus de Santa Apolónia, Bragança, Portugal

**Keywords:** hydrocolloid sources and derivatization, antiviral mechanisms of action, susceptible viruses, structure-activity relationship, complementary therapeutic strategies

## Abstract

Viruses remain a major global health challenge due to their strict dependence on host cell machinery and limited therapeutic options. Hydrocolloids (natural and semisynthetic) have gained attention as promising scaffolds for antiviral drugs discovery. Their structural variability, biocompatibility, and low toxicity enable diverse mechanisms of action, including inhibition of viral attachment and entry, disruption of replication, immunomodulation, and in some cases direct virucidal effects. This review examines the antiviral activity of hydrocolloids from three main sources: algal (agar, alginate, carrageenan, fucoidan, laminarin, and ulvan); animal (chitin, chitosan, chondroitin sulphate, dermatan sulphate, keratan sulphate, heparin, heparan sulphate, glycogen, and hyaluronan); and plant (pectin derivatives, starch derivatives, and locust bean gum). Across these groups, antiviral efficacy is strongly modulated by structural determinants such as molecular weight, degree and distribution of sulphation, glycosidic linkages, and branching patterns. Sulphated polysaccharides, in particular, exhibit broad-spectrum activity by blocking early infection steps through electrostatic interactions with viral proteins. Despite their potential, challenges persist, including structural heterogeneity, lack of viral specificity, and anticoagulant side effects in certain sulphated derivatives. Strategies to overcome these limitations include chemical modification, development of semisynthetic derivatives, and nanomaterial engineering to enhance stability, bioavailability, and therapeutic precision. Overall, hydrocolloids represent a versatile and underexplored platform for antiviral therapeutics. Continued efforts toward structural optimization, mechanistic elucidation, and clinical translation are critical to unlock their full potential against current and emerging viral threats.

## 1 Introduction

Viruses are characterized by a relatively simple architecture, consisting of a protein capsid that encapsulates their genetic (either DNA or RNA) material, together with viral enzymes and, in some cases, an outer lipid envelope. Since they lack the biochemical machinery for autonomous replication, viruses rely entirely on the host cell’s system, making them mandatory intracellular parasites. This hinders the development of virucidal therapies, as effective therapeutic agents must selectively block viral processes without damaging host cells. Accordingly, molecules capable of interfering with one or more stages of the viral replication cycle, thereby halting the onset and/or progression of infection while guaranteeing selective toxicity toward the pathogen without harming host cells, are considered effective antiviral candidates (1).

Moreover, viruses are wide-spread etiological agents, and current antiviral therapies, although valuable, often face challenges such as rapid emergence of resistant strains, limited efficacy across viral families, and potential host toxicity, emphasizing the urgent demand for new antiviral strategies (2). This has created interest in naturally derived biomolecules that may act through broad-spectrum and multitarget mechanisms (3).

Among the potential sources of antiviral compounds, hydrocolloids have emerged as particularly promising candidates due to their high structural variability, wide-ranging biological functions, biocompatibility, and low toxicity. These polysaccharides have increasingly been investigated in their natural state as well as in chemically modified forms designed to enhance their inhibitory properties against clinically significant viruses (4). Their capacity to act through distinct mechanisms of action further highlights their potential as promising candidates as scaffolds to novel antiviral agents (5).

**Figure 1:**
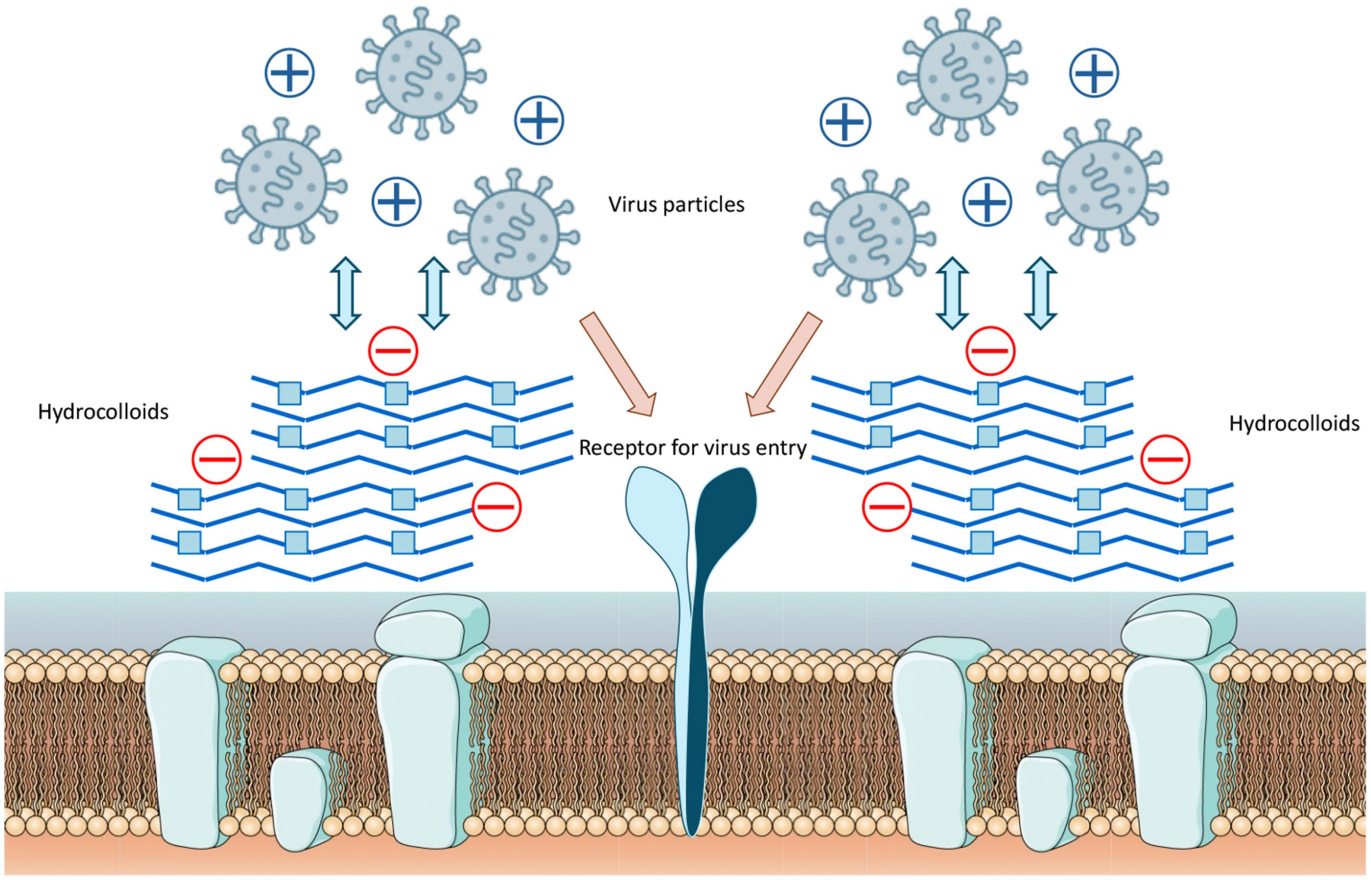
Mechanism of hydrocolloid-mediated viral inhibition by electrostatic interaction. Negatively charged sulphated groups (red circles) interact electrostatically with positively charged amino acids on viral surface proteins, preventing binding to host cell receptors and blocking entry.

Research has consistently demonstrated that the antiviral properties of hydrocolloids are strongly correlated to the presence of anionic groups, particularly sulphate moieties, which enable electrostatic interactions with viral envelope proteins and host cell receptors (Figure 1), preventing viruses from attaching to host cells and hindering the initial stages of viral infection (6). Moreover, there are additional structural determinants that modulate the antiviral activity of hydrocolloids:

(a) Glycosidic linkages - the type of glycosidic bond can determine the ability of hydrocolloids to inhibit viral enzymes (e.g., proteases), induce viral aggregation, or exert immunomodulatory effects. For instance, *β*-glucans, which feature β-(1 → 3) and β-(1 → 4) linkages, exhibit potent antiviral effects by enabling binding to immune receptors and stimulating immune responses (e.g., interferon production), leading to enhanced viral defence mechanisms. Conversely, *α*-glucans with α-(1 → 4) glycosidic linkages, such as starch, lack comparable antiviral activity (7).
(b) Molecular weight - generally, hydrocolloids with higher molecular weight display stronger antiviral effects, as they can form extended networks with viral particles and inhibit infection. High molecular weight hyaluronan, for instance, has demonstrated antiviral activity by blocking viral adhesion to host cells and interfering with the entry of enveloped viruses (7).
(c) Branching patterns - branched hydrocolloids often possess higher structural complexity, increasing their ability to disrupt viral processes. Fucoidans, for example, which are highly branched, exhibit notable antiviral activity by interfering with viral adhesion, entry, and fusion (8).

**Figure 2:**
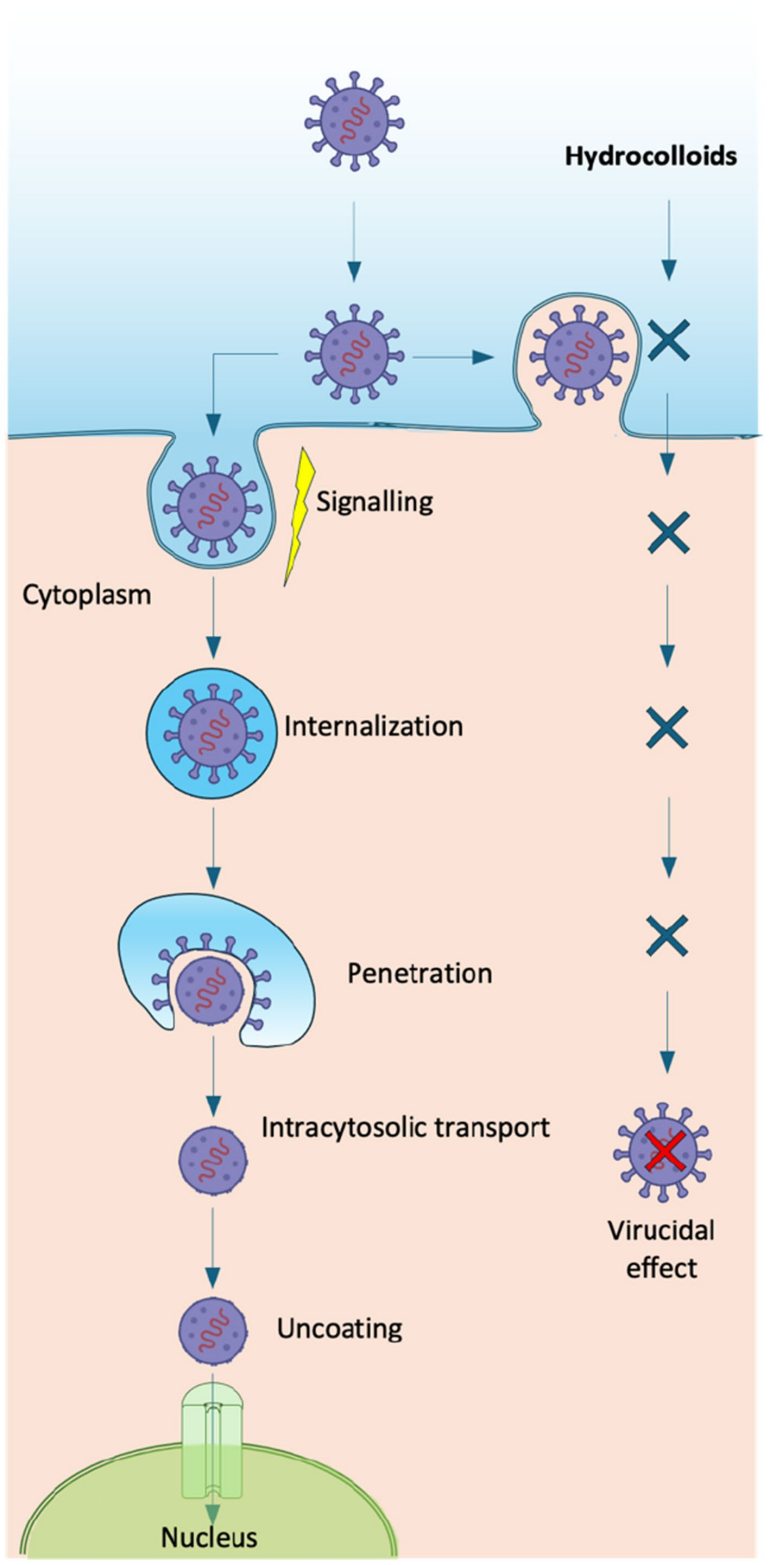
Viral infection pathway and inhibitory role of hydrocolloids. Hydrocolloids block viral attachment, entry, and replication steps, leading to a virucidal effect and preventing the release of the viral genome into the host cell nucleus.

**Figure 3:**
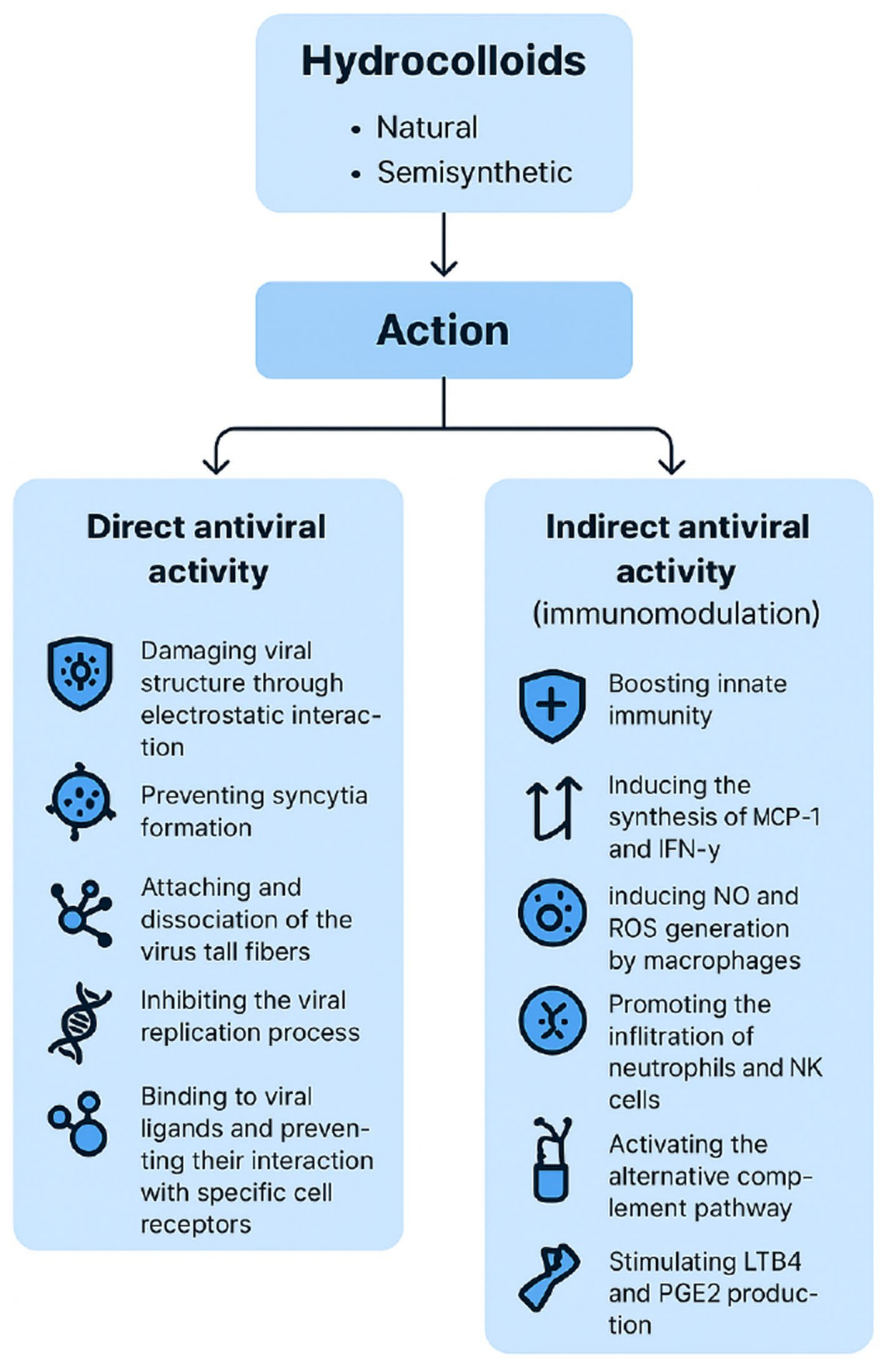
Main direct and indirect antiviral mechanisms of natural and semisynthetic hydrocolloids. Directly, hydrocolloids may block viral attachment and entry, inhibit viral replication, or inactivate viral particles, while is indirect action occurs mainly by modulating host immune responses. These mechanisms are influenced by structural determinants such as sulphation, branching, and molecular weight, which in turn affect clinical translation potential.

Overall, antiviral performance is modulated by parameters such as molecular weight, degree of sulfation, charge density, and conformational flexibility, which enable hydrocolloids to interfere with viral attachment and entry, stimulate immune responses, or directly inhibit replication (9). Likewise, chemical and/or enzymatic modifications (e.g., sulfation, carboxylation, or acetylation) can further strengthen these properties, producing semisynthetic derivatives with enhanced antiviral activity. Depending on these structural features, hydrocolloids may inhibit multiple stages of the virus cycle (Figure 2), including internalization, uncoating, and transcription phases, or even display direct virucidal effects (6). Different antiviral mechanisms (Figure 3) have been described:

(a) Blocking viral attachment and entry - sulphated polysaccharides, such as ulvan, fucoidan, or chondroitin sulphate, can effectively prevent viruses from binding and entering host cells during the initial stages of infection, while showing minimal toxicity at therapeutic doses (10).
(b) Inhibiting viral replication - several hydrocolloids (e.g., chitosan, heparin, ulvan, fucoidan, carrageenan) were shown to be capable of disrupting viral replication, as documented in studies involving herpes simplex virus types 1 and 2 (HSV-1 and HSV-2) and human immunodeficiency virus (HIV) (2).
(c) Immunomodulatory effects - some hydrocolloids (such as heparan sulphate, hyaluronan, alginate, carrageenan, fucoidan, laminarin, ulvan) modulate immune responses affecting lymphocyte proliferation, macrophage activity, inducing increased serum levels of immune-regulating cytokines (IL-2, TNF-*α*, and IFN-*γ*), or by altering spleen and thymus parameters (2).
(d) Direct viral inactivation - sulphated hydrocolloids such as alginate demonstrate the capacity to bind virus particles, inhibit early infection steps, and down-regulating host signaling pathways, effectively suppressing viral propagation while maintaining low cytotoxicity (11).

The former properties of hydrocolloids are particularly noteworthy, given the limited number of approved antiviral drugs, even in a scenario in which viral diseases are still causing significant morbidity and mortality (12). Consequently, screening hydrocolloids and other molecules with antiviral activity represents a valuable approach to discover new drug candidates and strengthening the available pool of antiviral treatments (13).

Additionally, and unlike small-molecule antivirals, hydrocolloids often exhibit heterogeneous structures that hinder reproducibility, standardization, and generalised use. Moreover, despite compelling *in vitro* evidence, translation into *in vivo* systems or clinical models is often inconsistent (1).

This review provides a critical overview of hydrocolloids of algal, animal, and plant origin, either in their native forms or as chemically modified derivatives. For each analysed hydrocolloid, the structural features, reported antiviral spectra, mechanisms of action, strengths, weaknesses and translational potential, are systematically described.

## 2 Hydrocolloids from algae sources

Algae are a rich source of structurally diverse hydrocolloids, several of which exhibiting notable antiviral activity, which is largely determined by sulphation patterns, molecular weight, and branching frequency. Below, six major algal hydrocolloids are compared regarding the former properties, being also evaluated for their strengths and limitations for therapeutic application.

### 2.1 Agar

#### 2.1.1 Structure

Agar consists primarily of two fractions: agarose and agaropectin. Agarose, corresponding to about 70% of the polysaccharide, is a linear polymer built from agarobiose (1,3-linked *β*-D-galactopyranose) and neoagarobiose (1,4-linked 3,6-anhydro-*α*-L-galactopyranose) units, which often contain alternating sulphate groups. Agaropectin, in contrast, accounts for around 30% of the mixture and is a more heterogeneous fraction, being characterized by a lower frequency of alternating D- and L-galactose residues with acidic side chains such as sulphate and pyruvate, or methoxy groups (9). Agarans, therefore, are commonly found in two fractions: neutral agarose and charged agaropectin, which are differentiated by their ionic composition (4).

#### 2.1.2 Occurrence and antiviral spectrum

The sulphate residues play a crucial role in mediating the antiviral activity, such as it has been observed with the sulphated agarans isolated from *Bostrychia montagnei* and *Acanthophora spicifera*, which demonstrated potent inhibition of HSV-1 and HSV-2, with the most active fractions being those enriched in sulphate (Table 1) (4). Beyond sulphated derivatives, agar itself has also shown antiviral potential. When incorporated into food coatings, it was able to reduce murine norovirus (MNV) infectivity, suggesting possible applications in food safety to mitigate the transmission of foodborne viruses (14). Agar was also shown to exert observable effect over plaque development, not only when tested against dengue virus (DENV), but also in assays conducted with encephalomyocarditis virus (ECMV) and Mengo encephalomyelitis virus (MEV), likely by blocking cell-virus interaction through immobilization of virus particles (15).

**Table 1:** Antiviral activity of natural and modified hydrocolloids from animal, algal, and plant sources.

Hydrocolloid structural units	Antiviral activity	Mechanism of action	Natural sources	References
Algae				
Agar 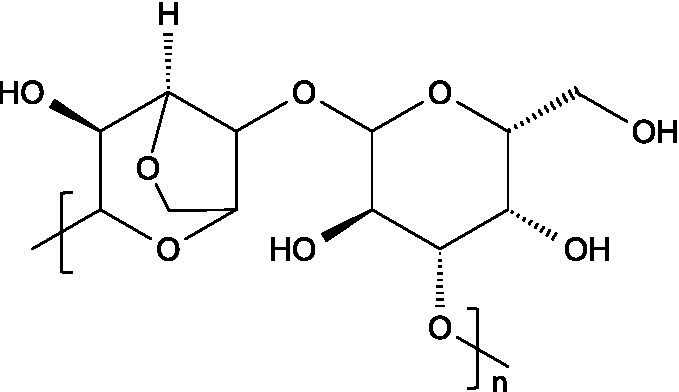	DENV, ECMV, HSV-1 and HSV-2, IAV, Mengo encephalomyelitis virus, MNV	Blocking viral attachment and entry. Electrostatic interaction. Interacting with viral glycoproteins and host cell surfaces. Modulation of plaque development.	*Acanthophora spicifera*, *Bostrychia montagnei*, *Gelidium cartilagenium*, *Gracilaria corticate*	(4, 9, 14, 15)
Alginate 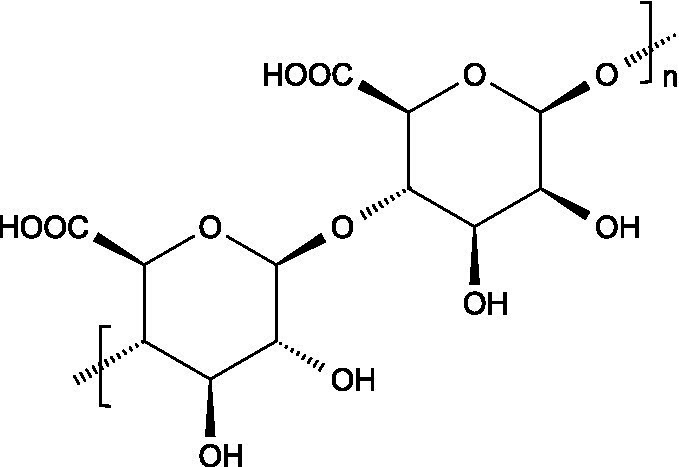	DENV, HBV, HCV, HIV-1, HPV, HSV-1, HSV-2, IAV, RV, SARS-CoV-2, SINV, TMV	Electrostatic interaction. Blocking viral adsorption. Binding to viral envelopes Interaction between the SARS-CoV-2 spike protein and the ACE2 receptor. Inhibition of SARS-CoV-2 3CLpro. Interacts with the RBD. Inhibiting DNA polymerase activity. Reducing reverse transcriptase. Preventing the binding of HIV-1 gp120 protein to T cells’ CD4 receptors. Stimulating the host immune response.	*Dictyota dichotoma*, *Padina pavonica*, *Sargassum cinereum*, *Sphacelaria indica*, *Turbinaria turbinata*	(2, 6, 17–22, 111)
κ-Carrageenan 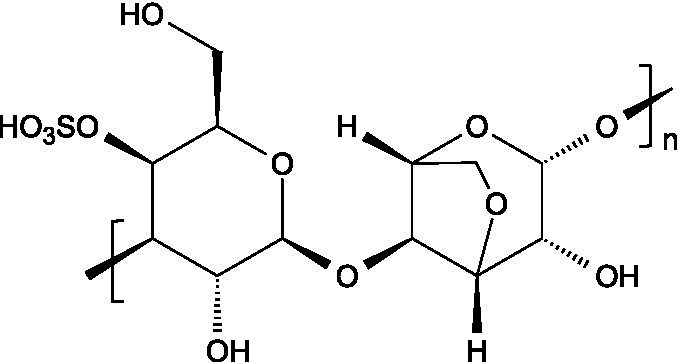	Avian sarcoma virus (ASV), Bovine herpesvirus type 1 (BoHV-1), CMV, DENV, ECMV, enterovirus, hepatitis A virus (HAV), HIV, HPV, HRV, HSV, IAV, murine CMV, RABV, RSV, simian foamy virus (SFV), SARS-CoV-2, SINV, TMV	Inhibiting viral adsorption, penetration, uncoating, replication and transcription. Direct virucidal effect. Blocking viral protein synthesis. Suppressing viral protein expression. Preventing glycoprotein-mediated fusion. Stimulating type I interferon production. Enhancing NK cell activity. Increasing secretion of IL-2 and TNF-α. Promoting lymphocyte proliferation.	*Euchema spinosum*, *Gigartina skottsbergii, Meristiella gelidium*	(2, 6, 21, 22, 26–30)
Fucoidan 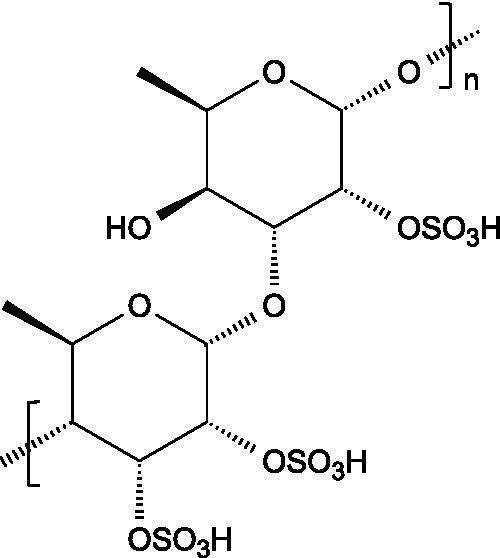	AIV, CVB-3, CMV, DENV, hPIV-2, HBV, HIV, HSV-1, HSV-2, IAV, NDV, SARS-CoV-2, VSV	Inhibiting viral fusion, internalization, and subsequent replication. Triggering the production of interferons and cytokines. Interfering with viral transcription and neuraminidase activity. Inhibiting 3CL^pro^ and binding to the viral spike S-glycoprotein in SARS-CoV-2. Shielding the positively charged amino acids within gp120. Inhibiting viral-induced syncytium formation. Increasing macrophage phagocytosis.	*Cladosiphon okamuranus*, *Cystoseira indica*, *Dictyopteris membranacea*, *Durvillaea antarctica*, *Fucus vesiculosus*, *Laminaria japonica*, *Padina pavonica*, *Padina tetrastromatica*, *Sargassum piluliferum*, *Turbinaria conoides*, *Undaria pinnatifida*	(6, 22, 36–41, 44, 111)
Laminarin 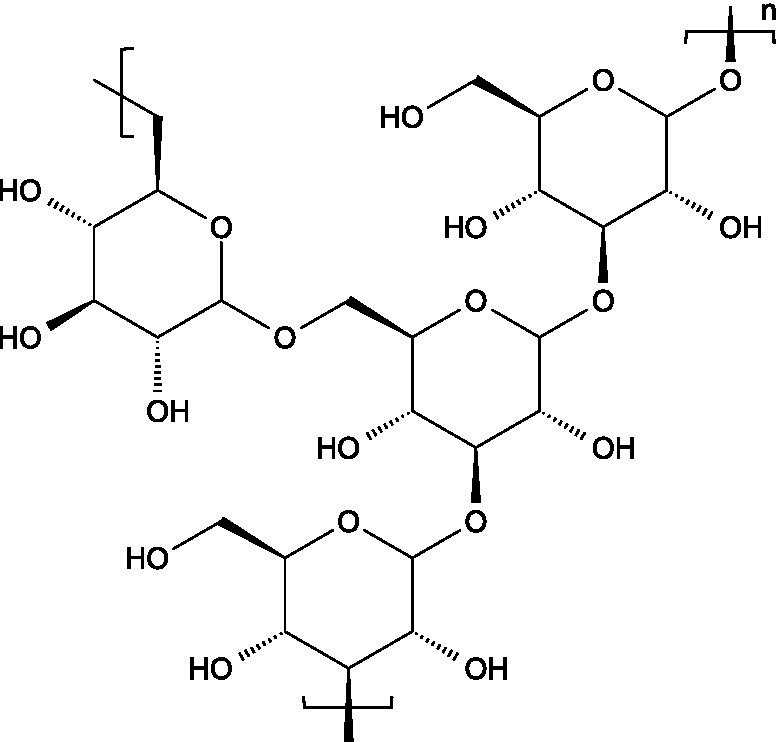	HIV, HSV-1, SARS-CoV-2, TMV	Inhibiting viral adsorption. Inhibiting reverse transcriptase. Enhancing the cGAS-STING pathway.	*Alaria esculenta*, *Ecklonia cava*, *Laminaria digitata*, *Laminaria hyperborea*, *Macrocystis pyrifera*, *Nereocystis luetkeana, Saccharina latissima*	(6, 111)
Ulvan 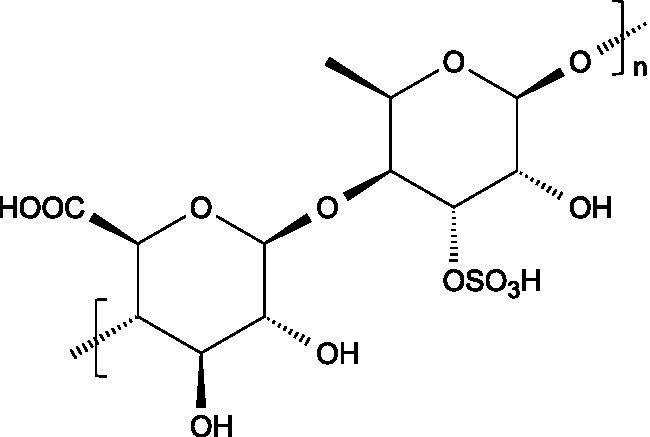	HSV-1, IFV-A, JEV, MeV, NDV, SARS-CoV-2	Electrostatic interaction of ulvan’s sulphated groups with viral surface proteins. Blocking replication. Preventing viral fusion. Blocking syncytia formation. Interfering with viral DNA replication and transcription. Downregulating protein synthesis. Decreasing the production of pro-inflammatory cytokines.	*Ulva clathrata*, *Ulva intestinalis*, *Ulva latuca*, *Ulva pertusa*	(6, 15, 30, 51, 54, 56, 112)
Animal				
Chitin 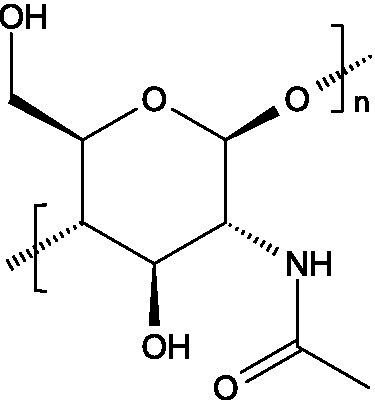 Chitosan 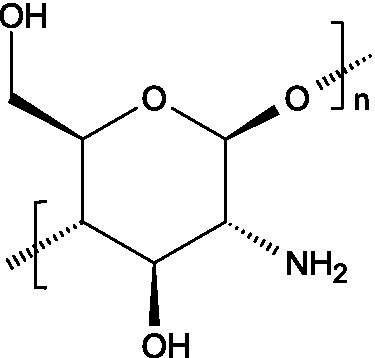	HIV-1, TMV, coronaviruses, HSV, adenovirus, H1N1 IAV, HPV, VSV, NDV, RVF and coxsackieviruses	Electrostatic interaction: (i) among the positively charged ChNC loaded with Zn^2+^ and the negatively charged TMV surface; (ii) among chitosan positive charges with the negative charges of capsid proteins facilitating the destruction of virus structure. Attaching to the virus tail fibres and causing virus dissociation. Interfering with the viral replication. Inhibiting ACE2 receptor. Attaching to the spike glycoprotein trimer cavity.	Crustacean shells	(58, 60, 61, 63)
Chondroitin sulphate 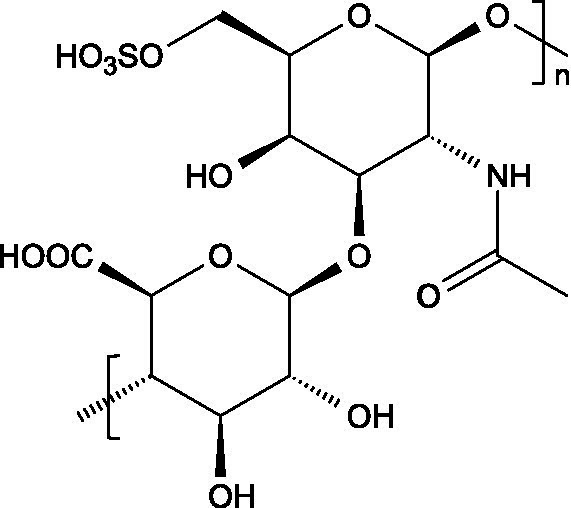	DENV, HIV, HSV, HTLV-1, IAV, SARS-CoV-2	Inhibition of adsorption and other early inhibition steps. Inhibition of SARS-CoV-2 M^pro^ activity. Blocking virus internalization. Acting as decoy receptor. Interfering with various points in the viral life cycle.	Pig, bovine, shark, and sea cucumber (*Thelenota anana*)	(6, 67, 68, 70)
Dermatan sulphate 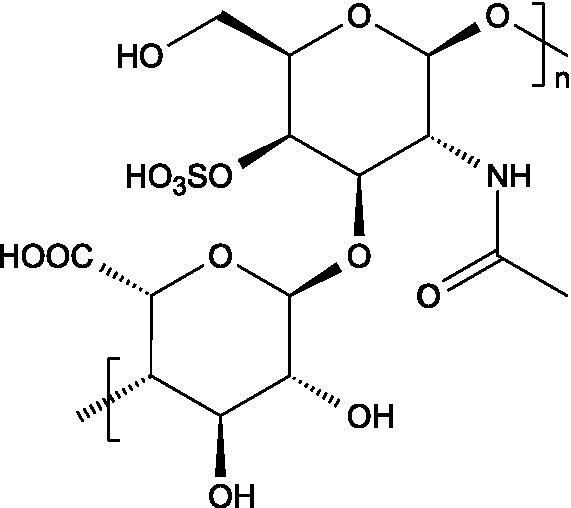	SARS-CoV-2	Mild activity when combined with fast moving heparin (sulodexide).	Shark skin, brittle stars, porcine intestinal mucosa	(72, 87, 109)
Keratan sulphate 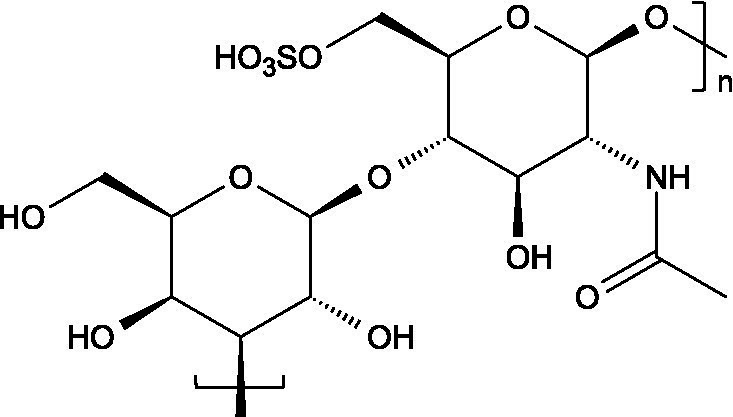	SARS-CoV-2	Acting as decoy receptor. Inhibiting the virus activity by binding to envelope ligand sites and blocking viral attachment.	Cartilaginous tissues, cornea, brain, and intervertebral discs.	(74)
Heparin 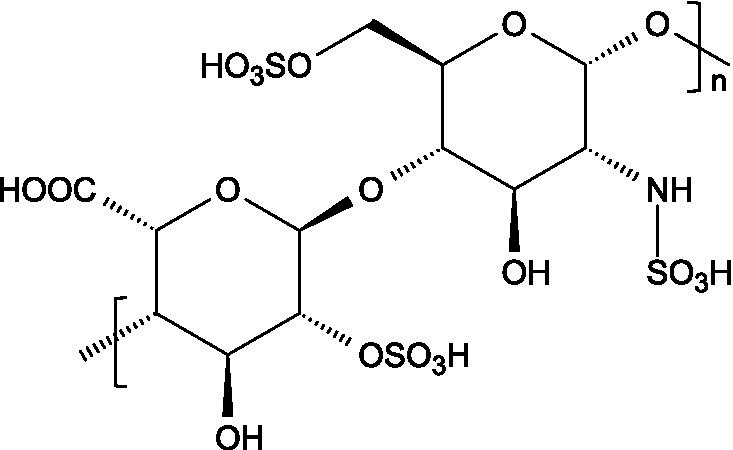	HSV-1, HSV-2, DENV, SARS-CoV-2, HPV, RABV, HIV-1, CVB-3, Zika virus	Prevents viral attachment and entry by blocking gC/gB binding. Potent inhibitor of E protein-mediated binding. High affinity binding to spike protein. Blocking gp120 binding. Blocking of virus-cell adhesion via heparin-like analogues. Preventing viral replication and promoting apoptosis of infected cells Early in vitro inhibition via sulfation-dependent mechanisms.	Bovine and porcine mucosa	(76–78, 80–83)
Heparan sulphate 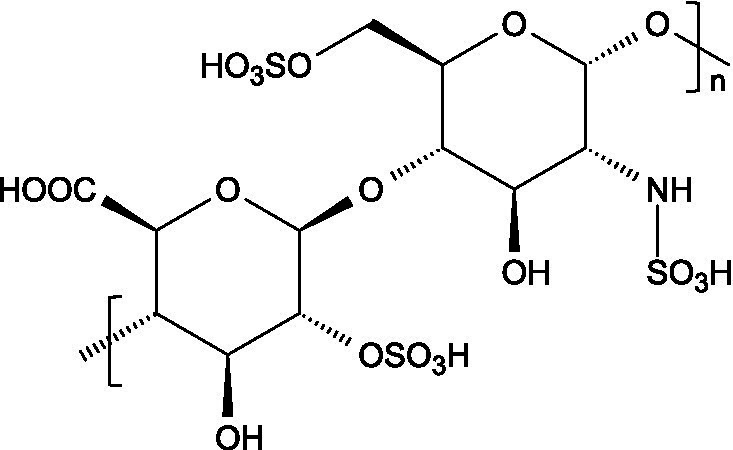	Several species of *Adenoviridae*, *Alphaviridae*, *Bunyaviridae*, *Caliciviridae*, *Conoronaviridae*, *Flaviviridae*, *Hepadnaviridae*, *Hepeviridae*, *Herpesviridae*, *Papillomaviridae*, *Paramyxoviridae*, *Parvoviridae*, *Picornaviridae*, *Polyomaviridae*, *Poxviridae*, *Retroviridae*, *Rhabdoviridae*	Acting as decoy receptor. Electrostatic interaction with viral glycoproteins. Triggering receptor-mediated endocytosis.	Bovine lungs, porcine intestinal mucosa.	(72, 73, 85)
Glycogen 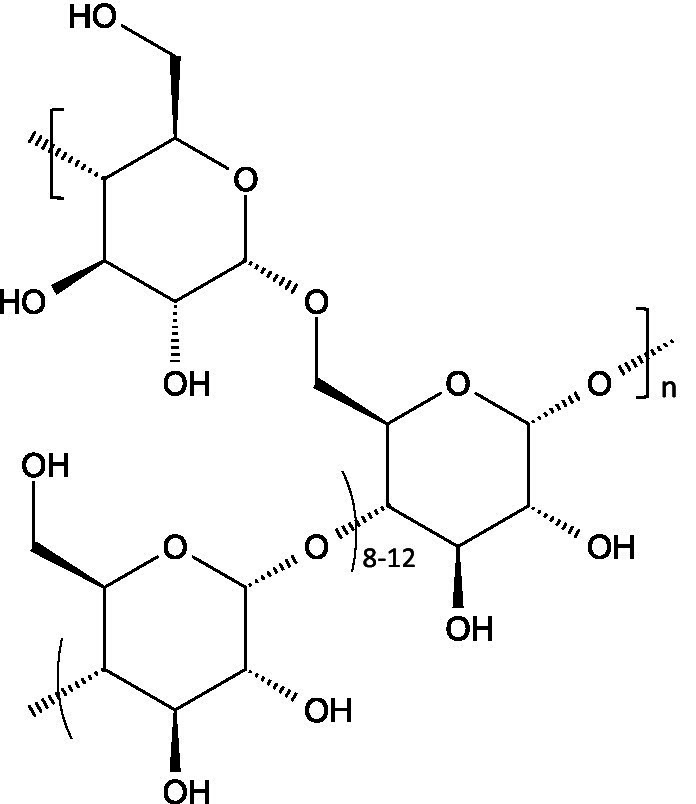		No antiviral activity was observed, although its sulphated form exhibited immunostimulatory activity by inducing the proliferation of lymphocytes and the production of antibodies.	Oysters	(89)
Hyaluronan 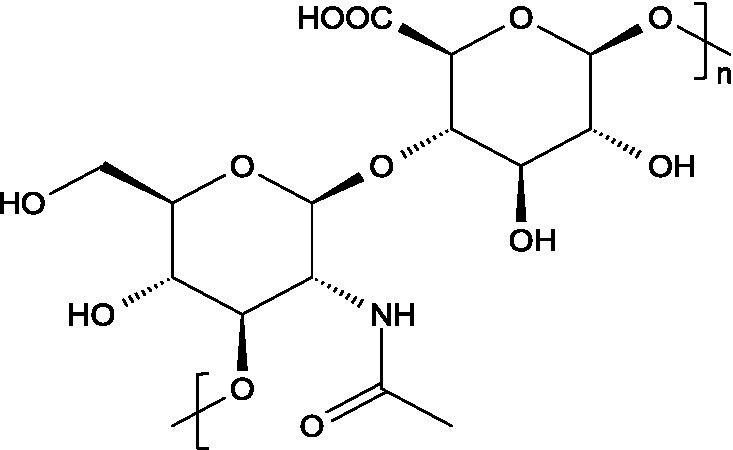	HSV-1, HSV-2, HIV	Reducing the infection of unstimulated CD4 + T helper cells in a CD44-dependent manner. Acting as decoy receptor. Inhibiting the virus activity by binding to envelope ligand sites and blocking viral attachment. Inhibiting the viral docking, internalization and uncoating in host cells. Improving the immune response of the host.	Bacteria, mammalian tissues	(15, 90, 92, 93, 110)
Plantae				
Pectin (derivatives) 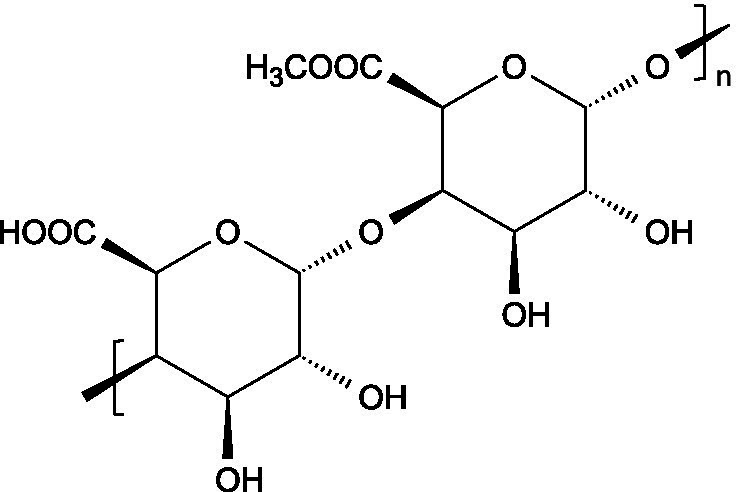	Enterovirus, HSV, poliovirus, RSV.	Interaction of the negatively charged sulphate groups with the positive charges in the viral envelope, leading to the formation of a high molecular weight sulphated pectin-virus complex that prevents the viral adsorption and penetration. Extracellular binding of sulphated pectin to the cellular receptors (initiating signaling pathways) or through intracellular interactions with effector molecules. Inhibition of the enzymes responsible for the genome replication (in maturation and release of viral particles stages).	*Cucumis melo*, *Dimorphandra gardneriana*, *Inga* spp., *Mangifera indica*, *Morinda citrifolia*, and *Salvia plebeia*	(10, 96–101)
Starch (derivatives) 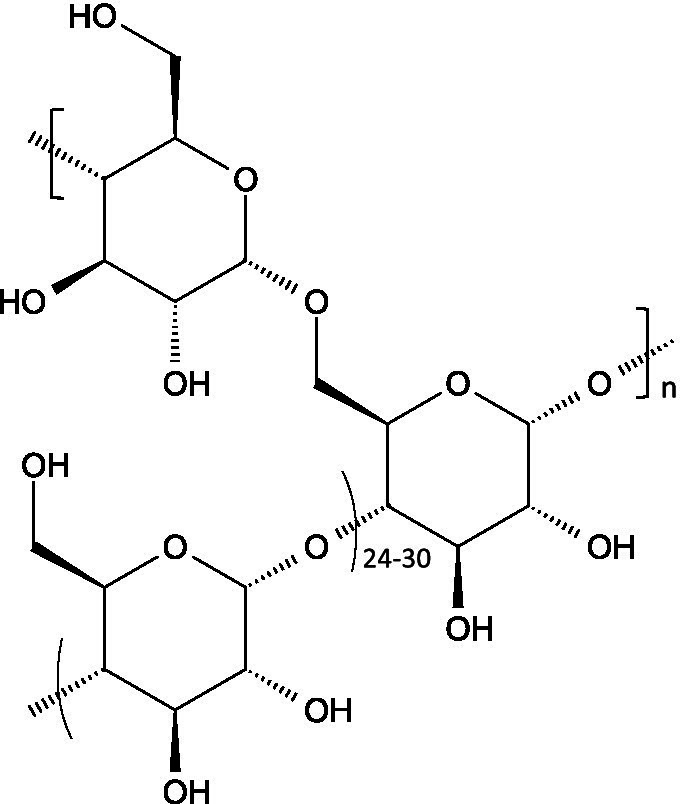	Coxsackievirus, MHV-3 coronavirus, SARS-CoV-2	Collapse of the phospholipid bilayer of the viral envelope.	*Manihot esculenta*	(103)
Locust bean gum 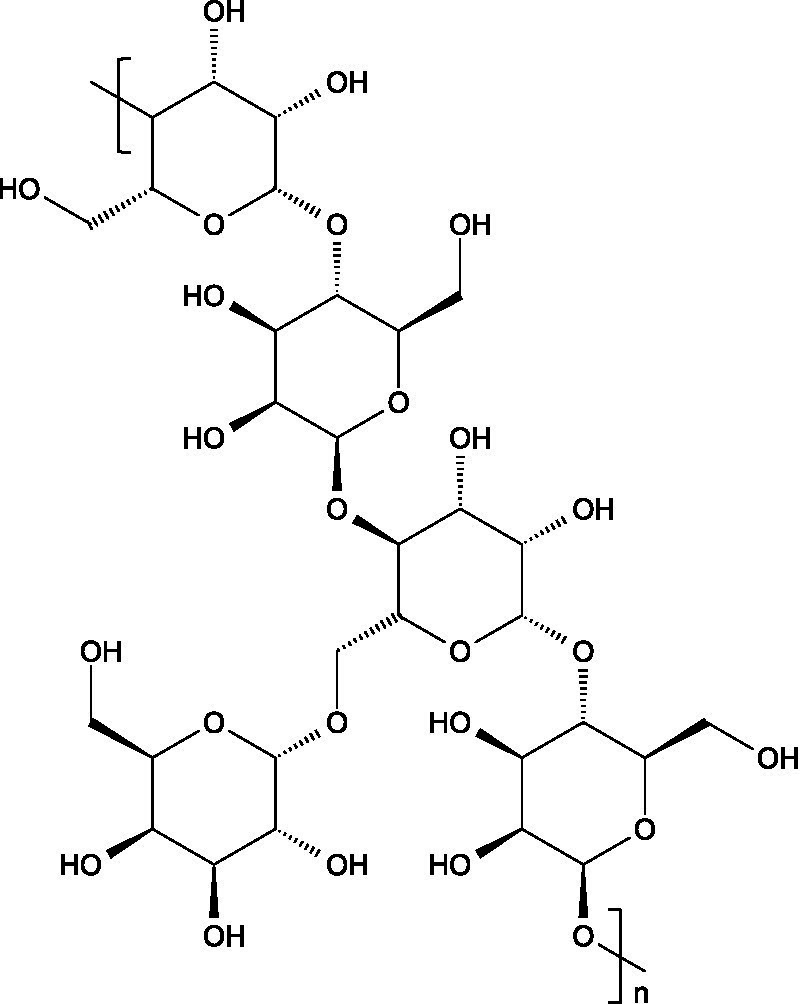	HIV, DENV	Blockage of internalization/uncoating. Electrostatic interaction with viral proteins.	*Ceratonia siliqua*	(108)

The susceptible viruses, main mechanisms of action, and natural sources of each hydrocolloid are summarized. Differences in structural determinants such as degree of sulphation, molecular weight, and branching are highlighted, as these strongly influence antiviral potency and potential for clinical translation.

#### 2.1.3 Mechanism of action

Activity is linked to sulphate content, which promotes electrostatic binding to viral envelope proteins and prevents host cell attachment. In the case of MEV, activity was verified to occur by blocking cell-virus interaction through immobilization of virus particles (15).

#### 2.1.4 Critical evaluation

Although agar derivatives demonstrate activity, they typically require higher concentrations than other algal polysaccharides to achieve measurable antiviral effects, raising concerns about their therapeutic relevance. Their greatest potential seems to be more associated to food safety applications such as antiviral coatings, translation into systemic therapy remains unlikely (4).

### 2.2 Alginate

#### 2.2.1 Structure

Alginates are hydrophilic linear polysaccharides found in the cell walls of brown algae that consist of *β*-D-mannuronic acid (M), and *α*-L-glucuronic (G) residues linked through 1,4-glycosidic bonds, organized into homopolymeric (poly-G, poly-M blocks) or heteropolymeric (MG blocks) arrangements (16).

#### 2.2.2 Occurrence and antiviral spectrum

Beyond their traditional industrial use in textiles and paper production, alginates are widely applied in food industry, cosmetics, and biomedical fields (mainly due to its polyanionic structure, which enable many of their biological properties, including antiviral activity). Several species of brown algae, including *Padina pavonica*, *Sargassum cinereum*, *Turbinaria turbinata*, *Sphacelaria indica*, and *Dictyota dichotoma*, have yielded alginates with activity (Table 1) against severe acute respiratory syndrome coronavirus 2 (SARS-CoV-2) - particularly noticeable in the alginates derived from *D. dichotoma*, against rubella virus (RV), HSV-1, HIV, and hepatitis B virus (HBV) and also, although in less extent, against influenza A virus (IAV), hepatitis C virus (HCV), and Sindbis virus (SINV) (14, 17–22).

#### 2.2.3 Mechanism of action

Their antiviral potential has been attributed to different mechanisms of action: electrostatic interactions with viral envelopes, inhibition of essential viral enzymes, interference with virus-receptor binding, and modulation of host immune responses (14, 17, 18). Prominently, one of the alginate-derived compounds tested against HIV was shown to block viral adsorption to host cells while also inhibiting reverse transcriptase activity (21).

#### 2.2.4 Critical evaluation

Overall, alginates display broad-spectrum activity, often through electrostatic interactions and enzyme inhibition, and are generally biocompatible and low in cytotoxicity, which is advantageous for clinical translation. However, their efficacy is inconsistent across viral families, and *in vivo* pharmacokinetics remain underexplored. Structural modifications enhance activity, but imply increased structural complexity, which may hinder reproducibility drug development (14).

### 2.3 Carrageenan

#### 2.3.1 Structure

Carrageenans are linear sulphated galactans consisting of alternating 1,3-linked *β*-D-galactopyranose and 1,4-linked *α*-galactopyranose residues. The three main structural types - *κ*, *ι*, and *λ* - differ in the number and position of sulphate ester substituents and in the presence of 3,6-anhydro-D-galactopyranose units: κ-carrageenan carries a single sulphate ester group (25–30%), ι-carrageenan contains two (28–30%), while *λ*-carrageenan has three (32–39%) (23, 24).

#### 2.3.2 Occurrence and antiviral spectrum

Carrageenans have demonstrated broad-spectrum antiviral properties (Table 1). For instance, carrageenans isolated from *Gigartina skottsbergii* effectively inhibited the transmission of human papillomavirus (HPV), while also suppressing HSV-1 and HSV-2 and human rhinovirus (HRV) (22, 25). Similarly, carrageenan derived from *Meristiella gelidium* inhibited HSV activity *in vitro* murine cytomegalovirus (CMV) *in vivo* (5).

Likewise, low-molecular-weight κ-carrageenan has been reported to inhibit IAV, HSV-2 and HPV16, respiratory syncytial virus (RSV) (26–30), SARS-CoV-2 (31), and HIV (22).

Moreover, *λ*-carrageenan from the red alga *Gigantina skotsbergii* exhibited significant inhibitory effects on rabies virus (RABV), SARS-CoV-2 and IAV (32), while *ι*-carrageenan from *Euchema spinosum* has been demonstrated to neutralize SARS-CoV-2 in a concentration-dependent manner, thereby reducing both transmission and progression of infection into the lower respiratory tract (33). In addition, both ι- and λ-carrageenan inhibited DENV replication in Vero and HepG2 cells (21).

#### 2.3.3 Mechanism of action

As with other sulphated hydrocolloids, both the degree of sulphation and the molecular weight are determinant factors for the antiviral activity of carrageenans. However, antiviral efficacy is not strictly proportional to the overall sulphation level; instead, the specific position and spatial arrangement of sulphate groups on the backbone are key factors for antiviral capacity (30). Carrageenans have been shown to act primarily by blocking the initial interactions between viral particles and host cell receptors, inhibiting its adsorption and internalization, and suppressing viral protein expression (25). Carrageenans also display immunomodulatory activity, such as stimulating type I interferon production, enhancing natural killer (NK) cell activity, increasing secretion of IL-2 and TNF-*α*, and promoting lymphocyte proliferation (21).

#### 2.3.4 Critical evaluation

Interestingly, carrageenan is one of the few hydrocolloids tested in clinical formulations, such as nasal sprays against respiratory viruses, besides displaying broad-spectrum activity. In turn, it holds potential anticoagulant effects and strain variability, which have produced mixed results in clinical translational trials, underscoring the need for controlled head-to-head studies (5).

### 2.4 Fucoidan

#### 2.4.1 Structure

Fucoidans represent a diverse group of sulphated fucans that are primarily composed of fucose (40–80%), sulphate esters, and uronic acids, along with smaller proportions of other polysaccharides such as alginate and laminarin (34). Among seaweed-derived polysaccharides, fucoidans are regarded as the most structurally heterogeneous, with high variation in both molecular structure and weight (100 to 5,200 kDa) depending on the species (34). Typically, fucoidan constitutes about 25–30% of the dry biomass of brown algae. Its backbone generally consists of *α*-(1 → 3)-linked L-fucopyranose units or alternating α-(1 → 3) and α-(1 → 4)-L-fucopyranosyl residues. These chains can be further substituted with sulphate or acetate groups and may contain glycosyl side groups such as glucuronic acid, in addition to minor amounts of monosaccharides including D-xylose, D-galactose, D-mannose, and uronic acids (35).

#### 2.4.2 Occurrence and antiviral spectrum

These structurally complex sulphated polysaccharides from brown algae, are well known for their strong anti-inflammatory properties and broad antiviral activity (Table 1), which extends to both RNA and DNA viruses, such as IAV, HSV, HBV, HIV, and vesicular stomatitis virus (VSV) (22, 36). Extracts from *Undaria pinnatifida* and *Kjellmaniella crassifolia* were shown to attenuate the effects of IAV by interfering with viral transcription and neuraminidase activity. Fucoidans from *Fucus vesiculosus* and *Laminaria japonica were reported to inhibit* parainfluenza virus type 3 and avian influenza virus (AIV) infection, respectively (21), while that derived from *Cladosiphon okamuranus* suppressed human parainfluenza virus type 2 (hPIV-2) infection in LLC-MK₂ cells (37). Moreover, fucoidans derived from *Padina tetrastromatica* and *Turbinaria conoides* were found to significantly reduce SARS-CoV-2 replication in host cells by inhibiting 3CL^pro^ (up to 97%), although without affecting RBD-spike binding (RBD) (38). Likewise, sulphated fucoidans from *Dictyota mertensii*, *Lobophora* var*iegata*, *Fucus vesiculosus*, and *Spatoglossum schroederi* inhibited HIV replication (39), while those extracted from *Padina pavonica*, *Dictyopteris membranacea*, and *Fucus evanescens*, were active against HSV-1, HSV-2 and coxsackievirus B3 (CVB-3) (40–42).

#### 2.4.3 Mechanism of action

Fucoidans exert their antiviral activity by binding to viral and host proteins, thereby inhibiting viral fusion, internalization, and subsequent replication. Again, the degree of sulphation and branching pattern strongly influence their antiviral efficacy. In addition, fucoidan can also display immunomodulatory effects, such as inducing interferons and cytokines, further supporting host antiviral defence (43). Additional studies demonstrated that fucoidan can also bind to the viral spike S-glycoprotein, thereby preventing SARS-CoV-2 entry into host cells (44), being also active against HIV, either by inhibiting reverse transcriptase (39), or by shielding the positively charged amino acids within gp120 (the viral envelope glycoprotein) through interactions with specific sulphate groups (21).

#### 2.4.4 Critical evaluation

In general, fucoidans exhibit remarkable diversity and strong activity against influenza, HIV, and coronaviruses. However, their structural complexity is a double-edged sword: while varied sulphation enhances antiviral potency, it hinders reproducibility and standardization. Furthermore, in most cases, human pharmacokinetic and safety data are limited, slowing its clinical progress (5).

### 2.5 Laminarin

#### 2.5.1 Structure

Laminarin, sometimes referred to as laminaran, is a storage glucan naturally present in brown algae. It typically consists of 20–25 glucose residues linked by *β*-(1 → 3) glycosidic bonds, with occasional β-(1 → 6)-linked side chains. The branching degree can vary depending on environmental factors and the harvesting period of algae (45). There are two types of polymeric chains within laminarin: M-type chains, which terminate with a mannitol residue at the reducing end, and G-type chains, which end with a glucose unit (46).

#### 2.5.2 Occurrence and antiviral spectrum

Laminarin is commonly extracted from species belonging to the *Eisenia*, *Laminaria*, *Sargassum*, and *Saccharina* genera, and has been associated with diverse antiviral activities (Table 1). For example, laminarin extracted from kelp was active against HIV, HSV-1 tobacco mosaic virus (TMV) (6, 15, 47, 48).

#### 2.5.3 Mechanism of action

Laminarin demonstrated capacity to inhibit both the adsorption and the reverse transcriptase of HIV (6), as well as capacity to enhance the cyclic GMP-AMP synthase stimulator of interferon genes (cGAS-STING), boosting the production of type I interferons in both human and murine cells in its action against viruses such as HSV-1 (48). Interestingly, this effect was markedly reduced in acetylated derivatives of laminarin, as confirmed by viral plaque assays and by analysing changes in viral DNA and protein synthesis (49).

#### 2.5.4 Critical evaluation

In comparison to other hydrocolloids, laminarin stands out for enhancing host immune pathways, offering a unique immunomodulatory advantage. However, antiviral activity is relatively narrow in scope and often attenuated in chemically modified forms, potentially indicating a limited therapeutic relevance (50).

### 2.6 Ulvan

#### 2.6.1 Structure

Ulvans are the major water-soluble polysaccharides present in the cell walls of green seaweeds. These heteropolysaccharides are characterized by a high degree of sulphation and a complex composition that includes L-rhamnose-3-sulphate, together with several neutral sugars such as xylose, glucose, mannose, galactose, arabinose, and structural polysaccharides like cellulose, xyloglucan, and glucuronan. Ulvans also contain uronic acids like glucuronic acid or iduronic acid (51).

Ulvan’s backbone usually consists of repeating disaccharide units of *α*-L-rhamnose-3-sulfate linked (1 → 4) to *β*-D-glucuronic acid (ulvano-biuronic acid A) or α-L-rhamnose-3-sulfate linked (1 → 4) to α-D-iduronic acid (ulvanobiuronic acid B); additionally, it also contains aldobiose fragments known as ulvanobioses (52).

#### 2.6.2 Occurrence and antiviral spectrum

Experimental studies have confirmed its activity against several viruses. For example, ulvan from *Ulva pertusa* was shown to block replication of VSV (15, 53), while ulvan isolated from *Ulva clathrata* inhibited Newcastle disease virus (NDV) *in vitro* by preventing viral fusion (6). Likewise, ulvan from *Ulva latuca* demonstrated strong inhibitory activity against SARS-CoV-2 (54, 55), while that from *Ulva intestinalis* was active against measles virus (MeV), HSV-1, and Japanese encephalitis virus (JEV) (6, 21, 30, 56).

#### 2.6.3 Mechanism of action

The antiviral effects (Table 1) of ulvan are generally linked to its negative charge, which enables interactions with viral surface proteins and thereby interferes with the early stages of viral infection, particularly viral adsorption and entry. These inhibitory properties are also influenced by the molecular weight of the polysaccharide (51). The inhibitory activity against SARS-CoV-2 was demonstrated to occur through multiple mechanisms: electrostatic interaction of ulvan’s sulphated groups with viral surface proteins (hindering viral adsorption), suppression of viral replication, and direct virucidal effects (54, 55). Ulvan also showed capacity to block syncytia formation (e.g., against MeV), interfere with viral DNA replication and transcription and downregulating protein synthesis (such as observed with HSV-1), and blocking virus adsorption by effectively decreasing the production of pro-inflammatory cytokines in JEV-infected glial cells (6, 21, 30, 56).

#### 2.6.4 Critical evaluation

Ulvan’s multi-targeted activity (blocking entry, replication, and immune modulation) is promising. However, antiviral effects vary widely across species and extraction methods, hampering reproducibility. Furthermore, *in vivo* studies are essential before considering it for clinical translational (4).

## 3 Hydrocolloids from animal sources

Animal-derived hydrocolloids, particularly glycosaminoglycans, are well-characterized regarding their structural features, having also been extensively studied for their interactions with viral proteins. Yet, their potential as antivirals is balanced by constraints such as their anticoagulant activity and variability induced by their natural source.

### 3.1 Chitin and chitosan

#### 3.1.1 Structure

Chitin is a linear polymer of *N*-acetyl-D-glucosamine (2-acetylamino-2-deoxy-D-glucose) and D-glucosamine (30), presenting a molecular weight that may vary among 1,030 and 2,500 kDa. Chitosan, in turn, consists of *β*-(1 → 4)-linked units of 2-acetylamino-2-deoxy-β-D-glucose and 2-amino-2-deoxy-β-D-glucose, reaching lower molecular weights (usually between 100 and 500 kDa) (57). Chitosan is also characterized by a lower degree of acetylation and for lacking inter-sheet hydrogen bonds, making it more hydrosoluble than chitin. Likewise, chitosan is soluble at pH values below 6.5, as the protonation of amino groups imparts polycationic characteristics (58).

#### 3.1.2 Occurrence and antiviral spectrum

Chitin is typically found in the exoskeletons of crustaceans and insects, though it can also be obtained via fungal fermentation processes (30); chitosan is obtained by deacetylation of chitin found in crustaceans exoskeletons (59). They’ve both demonstrated capacity to fight viral infections (Table 1), as demonstrated against TMV, HIV-1, HSV, Friend murine leukaemia helper virus (F-MuLV), adenovirus (in NIH-3 T3 cells), IAV H1N1, HPV, VSV, NDV, SARS-CoV-2, Rift Valley fever virus (RVFV) and coxsackieviruses (assayed in Vero cells) (60, 61).

#### 3.1.3 Mechanism of action

Chitin and chitosan can exert their activity by directly interacting with viruses, or by indirectly stimulating antiviral immune responses. Their direct antiviral action often involves electrostatic interactions between their cationic derivatives and the negatively charged viral envelope proteins, damaging viral structures, inactivating viruses after attaching to their tail fibres, or interfering with the viral replication process (60). Their sulphated derivatives blocked the interaction of HIV-1 gp120 with the CD4 receptor in cultured T cells, in addition to hinder viral uptake and cellular penetration (as observed with HSV-1 and F-MuLV) (58). In turn, the N-(2-hydroxypropyl)-3-trimethylammonium chitosan chloride (HTCC) derivative demonstrated activity against several coronaviruses, most likely by inhibiting their interaction with the angiotensin-converting enzyme 2 (ACE2) receptor (58), while quaternized chitosan derivatives were shown to destabilize viral surface proteins by interacting with viral capsid/envelope proteins (62).

Chitin was also used to produce chitin nanocrystals (ChNC), rod-shaped nanostructures with a high aspect ratio (much longer than wide) and surface carboxyl groups that enhance chelation and adsorption. When loaded with Zn^2+^, ChNCs significantly reduced viral infection, most likely due to electrostatic interactions between the positively charged Zn^2+^ on ChNCs and the negative charges on virus’ surface (63). In addition, chitosan was reported for preventing viral spread in plant tissues, by specifically activating resistance gene expression and eliciting defence responses (60).

#### 3.1.4 Critical evaluation

Chitosan’s tunability through chemical modification is its greatest strength, enabling tailored derivatives with improved potency. However, their efficacy *in vivo* is significantly conditioned by the lack of standardized formulations, which hampers systematic *in vitro* studies and further clinical translation (64).

### 3.2 Glycosaminoglycans

Research into proteoglycans began in the early 20th century, with work focusing on “chondromucoid” from cartilage and anticoagulant extracts such as heparin from liver tissue. Between 1930 and 1960, major progress was made in identifying and characterizing the polysaccharide components of these substances (then often called mucopolysaccharides). That period led to the elucidation of different structures, such as hyaluronan, dermatan sulphate, keratan sulphate, the isomeric forms of chondroitin sulphate, heparan sulphate, and heparin. Collectively, these polysaccharides were renamed as glycosaminoglycans to emphasize the presence of amino sugars and related carbohydrate units arranged in polymeric chains. Subsequent studies revealed how these chains attach to core proteins within proteoglycans. Glycosaminoglycans are major components of animal connective tissues, and their sulphated derivatives are usually associated with significant antiviral activity (65).

#### 3.2.1 Chondroitin sulphate

##### 3.2.1.1 Structure

The basic unit of this complex glycosaminoglycan consists of a repeating disaccharide of glucuronic acid and *N*-acetyl-galactosamine linked by *β*-(1 → 3) bonds. These disaccharides are interconnected by β-(1 → 4) glycosidic bonds, forming a linear chain that reaches a molecular weight ranging between 5 and 50 kDa (66).

##### 3.2.1.2 Occurrence and antiviral spectrum

Together with chitosan, chondroitin sulphate and heparin, two of the glycosaminoglycans described herein, are among the most investigated antiviral animal polysaccharides. Chondroitin sulphate (Table 1) is has shown potent inhibitory activity against HSV-1, DENV (demonstrated in Vero and BHK-21 cell lines) (67), SARS-CoV-2, and bovine coronavirus (BCoV) (68). Moreover, two fucosylated chondroitin sulphates isolated from the sea cucumbers *Isostichopus badionotus* and *Pentacta pygmaea* demonstrated strong activity against SARS-CoV-1 and SARS-CoV-2, as, to a lesser extent, against Middle East respiratory syndrome coronavirus (MERS-CoV), highlighting the potential of structurally modified derivatives as broad-spectrum antiviral agents (69).

##### 3.2.1.3 Mechanism of action

The acknowledged effects of chondroitin sulphate include blocking virus internalization, acting as decoy receptors, and interfering with multiple stages of the viral life cycle. In all cases, the effectiveness is highly correlated with the density of sulphate groups and molecular weight (6). In some cases, such as observed with flavivirus, the mechanism of action appears to depend on specific carbohydrate determinants within the polysaccharide backbone, which may function as critical epitopes for the interaction with the virus (67). More recently, the mechanism of action against SARS-CoV-2 was also clarified: chondroitin sulphate can act through competitive inhibition of the viral main protease (M^pro^), a key enzyme in the coronavirus replication cycle (70).

##### 3.2.1.4 Critical evaluation

In general, chondroitin sulphate demonstrates promising antiviral activity, but the strong source-dependent variability (e.g., pig, bovine, shark, sea cucumber) complicates the reproducibility. Likewise, additional research is necessary to consistently define the structure-activity relationship.

#### 3.2.2 Dermatan sulphate

##### 3.2.2.1 Structure

Like chondroitin sulphate, dermatan sulphate is composed of approximately equimolar proportions of hexosamine, acetyl groups, uronic acids, and sulphate groups. Both glycosaminoglycans share *N*-acetyl-D-galactosamine (GalNAc) as building block, but they differ in the type of uronic acid: dermatan sulphate contains L-iduronic acid (IdoA), while chondroitin sulphate contains D-glucuronic acid (GlcA). In dermatan sulphate, the repeating disaccharide sequence is formed through alternating glycosidic linkages: *β*-(1 → 4) bonds connecting GalNAc to IdoA, and *α*-(1 → 3) bonds linking IdoA to GalNAc (71).

##### 3.2.2.2 Occurrence and antiviral spectrum

Regarding its viral activity (Table 1), there are no recent papers (published after 2020 till submission of this review) reporting remarkable antiviral activity of dermatan sulphate (therefore the Mode of action subsection was not included herein). Nonetheless, when combined with fast-moving heparin (a mixture commercially known as sulodexide, which is clinically used to prevent and treat vascular diseases), dermatan sulphate improved the clinical outcomes of COVID-19 patients (72).

##### 3.2.2.3 Critical evaluation

Evidence base is sparse, particularly in most recent years. The main translational potential of dermatan sulphate, although unlikely, may lie in combined therapies, such as the exemplified sulodexide, which has been explored in COVID-19 patients for anticoagulant and endothelial-protective effects.

#### 3.2.3 Keratan sulphate

##### 3.2.3.1 Structure

Keratan sulphate consists of repeating disaccharides units made up of D-galactose and *N*-acetylglucosamine-6-sulphate connected by β-(1 → 4) linkages. Unlike other glycosaminoglycans, keratan sulphate does not attach to the proteoglycan core protein through the typical tetrasaccharide linker. Instead, its three known forms are characterized by distinct attachment modes: in keratan sulphate I, the chains are linked by an asparagine residue through a complex *N*-glycan; in keratan sulphate II, the connection is through the *N*-acetylgalactosamine linked to serine or threonine; in keratan sulphate III, the linkage involves a mannose residue connected to serine or threonine (73).

##### 3.2.3.2 Occurrence and antiviral spectrum

Many viruses including IAV, HSV, HIV, and members of the Coronaviridae family, including SARS-CoV-2, use glycosaminoglycans as initial attachment factors to host cells, facilitating their entry. Because of this role, glycosaminoglycans are typically characterized by high bioactivity in result of their interaction with cytokines, cell receptors, and components of the extracellular matrix. However, keratan sulphate, which was identified more recently compared to other glycosaminoglycans and is therefore less extensively studied, has scarce evidence of antiviral activity; even so, in samples obtained from bovine cornea, keratan sulphate has shown moderate activity against SARS-CoV-2 (74).

##### 3.2.3.3 Mechanism of action

As observed in other glycosaminoglycans, keratan sulphate binds to viral surface proteins, interfere with the attachment and potentially blocking host-receptor interactions.

##### 3.2.3.4 Critical evaluation

Keratan sulphate is largely understudied when compared to other glycosaminoglycans. In-depth systematic studies are necessary to determine its therapeutic value.

#### 3.2.4 Heparin

##### 3.2.4.1 Structure

Heparin is a highly sulphated glycosaminoglycan composed by repeating sulphated disaccharide units with prevalence of 2-*O*-sulphate-*α*-l-iduronic acid and 6-*O*-sulfate-*N*-sulfate-α-D-glucosamine linked by a 1,4-glycosidic bond as the most common unit (75).

##### 3.2.4.2 Occurrence and antiviral spectrum

Heparin is typically derived from porcine intestinal mucosa or bovine lung. Beyond its well-established anticoagulant role, heparin has demonstrated broad-spectrum antiviral activity (Table 1). Early studies showed its activity against HIV, RABV, HSV-1, HSV-2, DENV-2, Zika virus, and human CMV (6, 76, 77).

##### 3.2.4.3 Mechanism of action

Heparin acts by inhibiting the viral attachment (binding specific viral glycoproteins such as HIV gp120 and SARS-CoV-2 RBD spike protein), inhibiting viral enzymes, or supressing virus replication and promoting apoptosis of the infected cells (76–79). Mechanistic insights into heparin’s activity against SARS-CoV-2 suggest multiple antiviral modes of action, such as (i) allosterically hindering spike-ACE2 interactions, (ii) competing with host heparan sulphate proteoglycans that serve as viral coreceptors, and (iii) preventing spike cleavage by furin, thereby reducing viral entry efficiency (80). Heparin also inhibits the main protease (M^pro^) of SARS-CoV-2, with low inhibition constant and (Ki < 7 nM) and half maximal inhibitory concentrations (IC_50_ < 8 nM) (81). It has been further shown to inhibit virus entry and replication in primary bronchial epithelial cells. Given that SARS-CoV-2 infection is initiated predominantly in the nasal epithelium, intranasal administration of heparin has been proposed as a potential strain-agnostic prophylactic strategy (82). Furthermore, its favourable skin permeability, suggests potential utility in topical or inhalation-based formulations, particularly on exposed areas and sites of primary viral entry, e.g., ACE2 rich epithelia of the eye (conjunctiva/lids), nasal cavity, and mouth (83).

##### 3.2.4.4 Critical evaluation

Among glycosaminoglycans, heparin is the most advanced toward clinical application. However, its anticoagulant activity poses major risks for systemic use, limiting the clinical applications to topical or localized formulations (1).

#### 3.2.5 Heparan sulphate

##### 3.2.5.1 Structure

Unlike most glycosaminoglycans, which are modified primarily through *O*-sulphation, heparan sulphate also undergoes *N*-sulphation. This structural feature is determinant for its biological activity, as the sulphation of hydroxyl and amino groups on the *N*-acetylglucosamine units determines how heparan sulphate interacts with proteins, cytokines, and growth factors, ultimately ruling its bioactive roles (73).

From a structural perspective, heparan sulphate is composed of repeating disaccharide units containing *N*-acetylglucosamine and glucuronic acid. This is closely related to heparin, except for the fact that heparin contains iduronic acid instead of glucuronic acid as the hexuronic component. Heparan sulphate chains are covalently linked to the protein core of the proteoglycan through a serine residue, which is connected by a characteristic tetrasaccharide linker, consisting of one xylose, two galactose units and one glucuronic acid residue (84).

##### 3.2.5.2 Occurrence and antiviral spectrum

Given its verified involvement in viral entry processes, heparan sulphate has been investigated as a potential antiviral target (Table 1). Several viruses, such as HIV-1, SINV, HPV, HSV, human CMV, JEV, DENV, SARS-CoV-2, and Zika virus, as well as members of *Alphaviridae*, *Flaviviridae*, *Papillomaviridae*, *Parvoviridae*, *Picornaviridae*, or *Retroviridae*, were described as using heparan sulphate as an attachment factor to promote the initial contact with host cells (61, 85).

##### 3.2.5.3 Mechanism of action

Heparan sulphate fragments were used to coat viral attachment proteins, thereby preventing them from binding to host cells. However, the development of such therapy is challenging, because the preparation of these fragments is demanding and there is a limited availability of this type of molecules from natural sources (85). When assayed against HIV, heparan sulfate demonstrated high-affinity binding to gp120, leading to an increased concentration of the virus at the cell surface and promoting interactions with CD4 and the co-receptors CCR5 and CXCR4, thereby acting as a competitive inhibitor of viral attachment. Heparan sulphate showed similar interaction with other viral envelope glycoproteins (e.g., HSV, Zika, SARS-CoV-2, and DENV), in all cases reducing infectivity by triggering receptor-mediated endocytosis through additional cellular receptors. A similar effect was observed in non-enveloped viruses such as HPV, where heparan sulphate interacted with the capsid protein L1, triggering conformational changes that promote binding to specific entry receptors and subsequent internalization through endocytosis (72, 86).

In either case, the sulphation pattern, particularly at the 4-*O* and 6-*O* of *N*-acetylglucosamine, have been shown to be crucial for the inhibitory effect of heparan sulphate (87).

##### 3.2.5.4 Critical evaluation

Heparan sulphate act as co-factor for the infection of a diverse array of RNA and DNA viruses, making it a potential target for broad-spectrum antiviral strategies. However, the structural heterogeneity remains a barrier to therapeutic development.

Among glycosaminoglycans, chondroitin sulphate stands out for its potent activity against HSV and SARS-CoV-2 protease; however, its interspecies variability (e.g., pig, shark, sea cucumber) has been hindering an effective clinical translation. In contrast, interest in the antiviral potential of dermatan and keratan sulphates has declined, likely because both compounds seem considerably weaker than heparin. By comparison, heparin and heparan sulphate remain the most promising candidates for clinical translational, with well-validated mechanisms of action; nonetheless, their anticoagulant side effects suggest that localized administration (e.g., intranasal) is preferable to systemic use (1).

### 3.3 Glycogen

#### 3.3.1 Structure

Glycogen is a highly branched glucose homopolysaccharide linked via *α*-1,4-glycosidic bonds, with branch points (usually every 8–12 glucose units) formed by α-1,6-glycosidic linkages.

#### 3.3.2 Occurrence and antiviral spectrum

Glycogen is one of the primary carbon and energy reserves in living systems. Despite its importance in metabolism, native glycogen lacks the specific structural features necessary for strong antiviral interactions and therefore exhibits limited activity against viral infections. In turn, chemically modified glycogen derivatives have been shown to exert a variety of biological activities, including immunomodulatory, antitumour, and antiviral activities (88), as demonstrated by its sulphated derivatives against CMV and DENV, as well as stimulation of lymphocyte proliferation (Table 1). In addition to sulphated derivatives, carboxymethylated and phosphorylated forms were also reported to possess potent immunostimulatory properties (89).

#### 3.3.3 Mechanism of action

Sulphated glycogen can attach to surface proteins, interfering with viral binding, in addition to enhancing immune response.

#### 3.3.4 Critical evaluation

In general, native glycogen is largely inactive, requiring extensive chemical modification to display antiviral effects, raising challenges for scaling-up and regulatory approval. In turn, its sulphated derivatives exhibit strong immunomodulatory potential, although it remains poorly characterized.

### 3.4 Hyaluronan

#### 3.4.1 Structure

Hyaluronan consists of alternating units of *β*-1,4-D-glucuronic acid and β-1,3-*N*-acetyl-D-glucosamine (90), presenting varying molecular weight depending on the tissue where it is located (91).

#### 3.4.2 Occurrence and antiviral spectrum

This non-sulphated glycosaminoglycan is an essential component of both extracellular and pericellular matrices of many tissues in the body, including the lungs, where it contributes to development, tissue homeostasis, and injury repair processes (92). It is the only glycosaminoglycan biosynthesized directly at the cell membrane and is not covalently attached to proteoglycans. The antiviral activity of hyaluronan (Table 1) has been demonstrated against a wide range of viruses, including HSV-1, HSV-2, HIV, NDV, VSV, RV, RABV, Ebola virus, Coxsackievirus B5 (COXB5), mumps virus and IAV (H1N1 and H3N2), while only mild activity has been observed against porcine parvovirus (90, 92, 93).

#### 3.4.3 Mechanism of action

Hyaluronan has shown capacity to exert antiviral activity by different mechanisms, such as acting as decoy receptor, limiting the infection of unstimulated CD4 + T helper cells in a CD44-dependent manner, binding to envelope ligand sites, or disrupting viral docking, internalization and uncoating in host cells; it has also been demonstrated to enhance the host immune response by stimulating the release of antiviral mediators (15, 90).

#### 3.4.4 Critical evaluation

Hyaluronan’s lack of sulphation avoids anticoagulants effects, which is a major safety advantage over heparin. However, its antiviral activity is less consistent across viral families, and, like other hydrocolloids, it stills lacking sufficient clinical studies. In turn, its excellent biocompatibility makes it an attractive candidate for topical or adjunctive applications.

## 4 Hydrocolloids from plant sources

Sulphated polysaccharides with antiviral properties are uncommon in higher plants; however, promising opportunities are emerging from the chemical modification of natural compounds (semisynthetic hydrocolloids). This generally involves a two-step approach, starting by isolating the targeted compound and further subjecting it to sulphation. Such chemically altered molecules present valuable alternatives for developing antiviral therapeutic agents. Sulphated glucans and xylomannans, for instance, have demonstrated strong antiviral efficacy, showing to be active against human CMV, HSV-1, HSV-2, RSV, IAV, and HIV-1 (94).

### 4.1 Pectin

#### 4.1.1 Structure

Pectin consists of a complex group of polysaccharides primarily composed of a backbone of *α*-(1 → 4) linked polygalacturonic acid units, intercalated with rhamnose residues, and presenting side chains containing neutral sugars. The carboxyl groups on the galacturonic acid units can be esterified with non-carbohydrate groups such as methyl or acetyl groups.

#### 4.1.2 Occurrence and antiviral spectrum

Pectin is predominantly located in the primary cell walls of plants, especially in soft non-woody tissues (95). Pectin extracted from mango (*Mangifera indica*) peels showed no activity against RSV, contrasting to its sulphated derivatives, which effectively inhibited RSV-induced cytopathic effects (in Hep-2 cells, a well-established model due to its permissiveness to viral infection) (96). In contrast, other natural pectins, such as those obtained from *Cucumis melo*, *Dimorphandra gardneriana*, *Inga* spp., *Morinda citrifolia*, and *Salvia plebeia*, showed antiviral activity in its native form, as reported against RSV, HSV-1 and poliovirus (97–100).

#### 4.1.3 Mechanism of action

Sulphated pectin demonstrated a strong inhibition of the initial stages of the replication cycle, which might be related to the electrostatic interaction of its polyanionic sulphate groups and the positively charged residues in the viral envelope, leading to the formation of high molecular weight sulphated pectin-virus complexes that block viral adsorption and penetration (6). Sulphated pectin can also inhibit viruses through extracellular binding to cellular receptors (triggering signaling pathways) or through intracellular interactions with effector molecules (101); likewise, it can affect the later stages of viral replication, such as maturation and release of viral particles, suggesting a potential interference with enzymes involved in genome replication (10).

#### 4.1.4 Critical evaluation

Sulphated pectins display strong activity at low micromolar concentrations with limited cytotoxicity, whereas native pectin is largely inactive. However, their translation into clinical uses still requires studies to validate their safety and stability *in vivo* (2).

### 4.2 Starch

#### 4.2.1 Structure

Starch consists of D-glucose units linked through *α*-(1 → 4) glycosidic bonds in linear chains (amylose) and α-(1 → 6) at branched points (amylopectin), usually separated by 24–30 glucose units (102).

#### 4.2.2 Occurrence and antiviral spectrum

Native starch is widely recognized as an energy source, and, in its native form, it generally lacks direct antiviral activity. However, starch derivatives obtained by chemical modifications, such as oxidation, carboxymethylation, phosphorylation, esterification, ozonation, and acetylation, have demonstrated antiviral properties (Table 1) (6, 103). More recently, a novel starch derivate prepared through nucleophilic substitution of hydroxyl groups with quaternary ammonium salts has been gaining attention as a promising therapeutic agent due to its antimicrobial properties (104). Actually, quaternized ammonium starch (QAS) achieved 99% viral inactivation rate after just 1 min of contact with the MHV-3 coronavirus (105).

#### 4.2.3 Mechanism of action

The capacity of starch derivatives to inactivate non-envelope viruses (such as coxsackievirus) was shown to occur by directly interacting and disrupting viral particles (6). In the case of QAS, the associated mechanism of action is attributed to electrostatic interactions between the positively charged quaternary ammonium groups and the negatively charged virus surface. As the quaternary amino groups in QAS have limited diffusion into the phospholipid bilayer of the virus, this leads to the disruption of the viral membrane and subsequent viral inactivation (106).

#### 4.2.4 Critical evaluation

Native starch is nearly inactive, whereas its derivatives exhibit relevant potential for topical or surface applications due to their fast-acting antiviral properties. However, their toxicological profile remains insufficiently characterized, limiting regulatory approval and precluding systemic use until safety is adequately established (103).

### 4.3 Locust bean gum

#### 4.3.1 Structure

Locust bean gum (LBG) is mainly composed by galactomannans consisting of a linear chain of (1 → 4)-linked *β*-D-mannopyranosyl units with (1 → 6)-linked *α*-D-galactopyranosyl side chains. The mannose and galactose contents range from 73–86% and 27–14%, respectively, resulting in a typical mannose:galactose ratio of about 4:1 (107).

#### 4.3.2 Occurrence and antiviral spectrum

The antiviral activity of sulphated derivatives of the galactomannan gum derived from the endosperm of *Ceratonia siliqua* (L.) Taub was evaluated against HIV using an MT-4 cell model. Sulphated LBG demonstrated an anti-HIV activity (Table 1) comparable to that of dextran and curdlan sulphate, two sulphated polysaccharides reported to have the strongest anti-HIV activity (0.1 to 2.4 μg/mL) combined with low cytotoxicity. Furthermore, this anti-HIV effect of sulphated LBG increased proportionally with its degree of sulphation (108). Sulphated LBG was also reported for its activity against DENV in an LLC-MK2 cell model, again in line with the anti-DENV effects observed for dextran and curdlan sulphate. As in the MT-4 cell assays, sulphated LBG showed no acute cytotoxicity in LLC-MK2 cells. The anti-DENV activity was also proportional to the degree of sulphation.

#### 4.3.3 Mechanism of action

The proposed mechanism of action is likely to occur via electrostatic interaction between the negatively charged sulphate groups of sulphated LBG and the positively charged amino groups of poly-L-lysine in viral proteins; independently of the mechanism, the demonstrated antiviral activity is associated with the molecular weight of sulphated LBG (108).

#### 4.3.4 Critical evaluation

Generally, locust bean gum sulphated derivatives stand out for inhibiting HIV and DENV at low concentrations, comparable to those presented by leading algal sulphated polysaccharides. However, its efficacy is highly dependent on the sulphation degree, which may complicate the reproducibility in therapeutic development.

## 5 Conclusion

Hydrocolloids, especially their sulphated derivatives, have emerged as promising antiviral agents with diverse mechanisms of action, including blocking viral attachment, interfering with entry and fusion, modulating host immune responses, suppressing of replication, and direct virucidal action. Their antiviral potential is strongly influenced by structural features such as degree and position of sulphation, molecular weight, branching patterns, and glycosidic linkages. In fact, a higher sulphation degree often enhances activity by increasing electrostatic interactions with positively charged viral proteins. Importantly, these structural determinants also provide opportunities for therapeutic optimization. For instance, selective *O*-sulphation has been shown to preserve antiviral potency while reducing anticoagulant effects, thereby widening the therapeutic window. Likewise, tuning molecular weight can balance viral binding affinity with improved tissue penetration and pharmacokinetics, while branching complexity may be harnessed to design semisynthetic mimetics that maximize electrostatic interactions without retaining unwanted biological activities.

Comparatively, algal polysaccharides such as carrageenan and fucoidan demonstrate broad-spectrum activity, with carrageenan already tested in clinical nasal sprays, though efficacy varies. Animal-derived glycosaminoglycans, particularly heparin, exhibit strong mechanistic evidence, but are constrained by anticoagulant risks. Plant-derived hydrocolloids generally require extensive modification to exhibit antiviral properties, entailing regulatory and toxicological challenges.

A critical evaluation shows that their clinical translation is hindered by structural heterogeneity, inconsistent virus-specific activity, and side effects such as anticoagulation. This requires efforts to design derivatives with strong antiviral effects but minimal side effects, or combination approaches with other polysaccharides or synthetic drugs. Importantly, the structural determinants that control antiviral activity also provide levers for therapeutic optimization. For example, selective *O*-sulphation can preserve antiviral potency while reducing anticoagulant effects, tuning molecular weight can improve tissue penetration, and branching modifications may enhance viral binding while minimizing toxicity. These strategies, combined with formulation approaches such as nanocarrier encapsulation or localized delivery (e.g., intranasal sprays), offer realistic pathways to overcome current limitations and translate hydrocolloids into clinically viable antivirals.

Likewise, and to move forward, future research must prioritize (i) comparative head-to-head evaluations to identify the most promising candidates; (ii) structural modifications that balance potency with safety; (iii) nanocarrier-based strategies to improve bioavailability; (iv) rigorous *in vivo* and clinical trials addressing dose, pharmacokinetics, and host-virus interactions.

Personalized medicine approaches, integrating insights from virology, immunology, and pharmacology, will also be critical to tailoring treatments to individual patient profiles.

Only by addressing these challenges hydrocolloids will evolve from promising laboratory findings to practical antiviral therapies.

## Glossary

GalNAc: *N*-acetyl-D-galactosamine
ACE2: angiotensin-converting enzyme 2
AIV: avian influenza virus
ASV: avian sarcoma virus
BCoV: bovine coronavirus
BoHV-1: bovine herpesvirus type 1
ChNC: chitin nanocrystals
CVB-3: coxsackievirus B3
COXB5: Coxsackievirus B5
cGAS-STING: cyclic GMP-AMP synthase stimulator of interferon genes
CMV: cytomegalovirus
DENV: dengue virus
ECMV: encephalomyocarditis virus
F-MuLV: friend murine leukaemia helper virus
GlcA: D-glucuronic acid
HAV: hepatitis A virus
HBV: hepatitis B virus
HCV: hepatitis C virus
HSV-1: herpes simplex virus type-1
HSV-2: herpes simplex virus type 2
HIV: human immunodeficiency virus
hPIV-2: human parainfluenza virus type 2
HPV: human papillomavirus
HRV: human rhinovirus
HTCC: N-(2-hydroxypropyl)-3-trimethylammonium chitosan chloride
MNV: murine norovirus
IdoA: L-iduronic acid
IAV: influenza A virus
JEV: Japanese encephalitis virus
LBG: locust bean gum
MeV: measles virus
MEV: Mengo encephalomyelitis virus
MERS-CoV: Middle East respiratory syndrome coronavirus
NDV: Newcastle disease virus
QAS: quaternized ammonium starch
RABV: rabies virus
RBD: receptor-binding domain
RSV: respiratory syncytial virus
RVFV: Rift Valley fever virus
RV: rubella virus
SARS-CoV-2: severe acute respiratory syndrome coronavirus 2
SFV: simian foamy virus
SINV;: Sindbis virus
TMV: tobacco mosaic virus
VSV: vesicular stomatitis virus
